# Fabrication and Characterization of Pectin Films Containing Solid Lipid Nanoparticles for Buccal Delivery of Fluconazole

**DOI:** 10.3390/ijms25105413

**Published:** 2024-05-16

**Authors:** Namon Hirun, Jongjan Mahadlek, Sontaya Limmatvapirat, Pornsak Sriamornsak, Etsuo Yonemochi, Takayuki Furuishi, Pakorn Kraisit

**Affiliations:** 1 Thammasat University Research Unit in Smart Materials and Innovative Technology for Pharmaceutical Applications (SMIT-Pharm), Faculty of Pharmacy, Thammasat University, Pathumthani 12120, Thailand; namon.hi@tu.ac.th; 2 Pharmaceutical Intellectual Center “Prachote Plengwittaya”, Faculty of Pharmacy, Silpakorn University, Nakhon Pathom 73000, Thailand; mahadlek_j@su.ac.th; 3 Pharmaceutical Biopolymer Group (PBiG), Faculty of Pharmacy, Silpakorn University, Nakhon Pathom 73000, Thailand; limmatvapirat_s@su.ac.th (S.L.); sriamornsak_p@su.ac.th (P.S.); 4 Department of Physical Chemistry, School of Pharmacy and Pharmaceutical Sciences, Hoshi University, Tokyo 142-8501, Japan; e-yonemochi@hoshi.ac.jp (E.Y.); t-furuishi@hoshi.ac.jp (T.F.)

**Keywords:** fluconazole, pectin, solid lipid nanoparticles (SLNs), buccal film, antifungal

## Abstract

Fluconazole (FZ) is a potential antifungal compound for treating superficial and systemic candidiasis. However, the use of conventional oral drug products has some limitations. The development of buccal film may be a potential alternative to oral formulations for FZ delivery. The present study involved the development of novel FZ-loaded solid lipid nanoparticles (FZ-SLNs) in pectin solutions and the investigation of their particle characteristics. The particle sizes of the obtained FZ-SLNs were in the nanoscale range. To produce pectin films with FZ-SLNs, four formulations were selected based on the small particle size of FZ-SLNs and their suitable polydispersity index. The mean particle sizes of all chosen FZ-SLNs formulations did not exceed 131.7 nm, and the mean polydispersity index of each formulation was less than 0.5. The properties of films containing FZ-SLNs were then assessed. The preparation of all FZ-SLN-loaded pectin films provided the mucoadhesive matrices. The evaluation of mechanical properties unveiled the influence of particle size variation in FZ-SLNs on the integrity of the film. The Fourier-transform infrared spectra indicated that hydrogen bonds could potentially form between the pectin-based matrix and the constituents of FZ-SLNs. The differential scanning calorimetry thermogram of each pectin film with FZ-SLNs revealed that the formulation was thermally stable and behaved in a solid state at 37 °C. According to a drug release study, a sustained drug release pattern with a burst in the initial stage for all films may be advantageous for reducing the lag period of drug release. All prepared films with FZ-SLNs provided a sustained release of FZ over 6 h. The films containing FZ-SLNs with a small particle size provided good permeability across the porcine mucosa. All film samples demonstrated antifungal properties. These results suggest the potential utility of pectin films incorporating FZ-SLNs for buccal administration.

## 1. Introduction

Fluconazole (FZ) is a synthetic triazole agent belonging to the therapeutic compound class of azole antifungals. The antifungal action of FZ arises from the depletion of fungal ergosterol synthesis and impairment of the fungal cell membrane [1]. FZ is a potential antifungal used to treat superficial and systemic candidiasis caused by a fungus of the *Candida* genus. Although oral tablets and suspensions of FZ are commercially available, the oral administration of FZ has some limitations associated with first-pass metabolism and side effects, such as stomach upset, diarrhea, nausea, and vomiting [2,3,4]. In addition, it has been suggested that the rapid dissolution of FZ from solid oral dosage forms may contribute to the increased chance of side effects [5]. Lipid-based nanocarriers represent a potential strategy for addressing limitations associated with traditional oral therapy [6]. In recent years, the fabrication of novel lipid-based nanocarriers has attracted increasing attention and has been considered a promising vehicle for delivering antifungals [3,7]. To ensure efficient drug delivery and avoid the risk of side effects, the incorporation of nanocarriers into dosage forms has been proposed for buccal delivery instead of conventional oral drug administration [8]. Therefore, the development of a suitable dosage form containing lipid-based nanocarriers for buccal administration is a promising approach for FZ delivery.

Solid lipid nanoparticles (SLNs), which typically range in size from 10 to 1000 nm in diameter, are colloidal lipid-based nanocarriers made up of a solid lipid core surrounded by a surfactant layer [7,9]. The structural characteristics and types of SLNs depend on the preparation techniques, the preparation parameters, the chemical nature of formulation components, and the composition of the formulation [6,10]. The main advantages of using SLNs to deliver active compounds are their ability to enhance absorption and increase safety [10]. Furthermore, the ability of the SLNs to sustain the release of the active compound and promote the relevant biological activity makes them promising lipid-based nanocarriers for a variety of applications [11]. Over the years, the fabrication of drug-loaded SLNs has gained attention to accomplish enhanced permeability for transdermal drug delivery [12]. In addition, SLNs have been considered as a potential platform to improve drug permeability through the buccal mucosa [13]. The buccal delivery of drugs from the site of application to the bloodstream can bypass first-pass metabolism, avoid pre-systemic inactivation in the gastrointestinal tract, and minimize gastrointestinal side effects [8,14]. Therefore, the buccal administration of SLNs offers a viable, non-invasive strategy for local and systemic therapy. However, the use of plain drug-loaded SLNs for buccal delivery faces a challenge due to their short residence time on the buccal mucosa. The clearance of SLNs from the buccal mucosa is a consequence of tongue and masticatory movements, dilution by saliva, and swallowing [14,15]. Recently, the strategy of incorporating SLNs into the mucoadhesive matrix, which can achieve prolonged adhesion on the mucosa surface, has become an attractive approach to overcome the limitation [15,16].

Mucoadhesion, the phenomenon of adhesion between material and mucous membrane, attracts considerable research attention that has been devoted to extending the residence time of pharmaceutical drug formulations at the mucosal surface [17,18]. Mucoadhesive preparations are capable of mucoadhesion to offer drug release at the target site and/or to facilitate drug permeation across the mucosa [19]. Several mucoadhesive dosage forms, such as tablets, discs, and films, have been explored for buccal administration [20]. Mucoadhesive films are often preferred over other dosage forms as buccal delivery systems. This is because of the high flexibility, appropriate thickness, and suitable softness of the films, which provide better patient compliance [20,21]. To achieve the mucoadhesive capability, the buccal mucoadhesive films are fabricated with mucoadhesive polymers used as major film formers [22]. The existing literature emphasizes that the mucoadhesion produced by the mucoadhesive polymers is derived from the interconnection between the mucus and the polymers through physical association (chain interpenetration and entanglement) and chemical interactions, mainly H-bonding and van der Waals forces [20,22,23]. The presence of H-bonding functional groups, including hydroxyl, carboxyl, amine, and amide groups, in the polymer structure contributes to adhesion to the mucosa [23]. In addition, the chain flexibility of the mucoadhesive polymeric component is anticipated to encourage mucoadhesion of the dosage forms [19,22].

Pectin is a natural heteropolysaccharide, the structure of which is rich in carboxylic groups. Although the structure of pectin is complex and possesses interlinked distinct domains, the main component of pectin is homogalacturonan residue, which is partially methyl-esterified. According to the degree of esterification (DE), pectin can be classified into two major groups: low DE pectins (DE < 50%) and high DE pectins (DE > 50%). High DE pectins with a high molecular weight have high mucoadhesive performance on the mucosa of the gastrointestinal tract, especially the tissues of the buccal, stomach, small intestine, and large intestine, because their molecular features enable the formation of H-bonds as well as great entanglements between polymer and mucin chains [23,24]. To constitute a film layer, pectins with high DE values have the potential to be utilized as film-forming material [25,26]. As the DE of pectins increased, the films became more hydrophobic [27]. The formulations made from pectin with high DE are superior to the more hydrophilic pectin-derived matrices in terms of the sustained release of entrapped molecules [27]. Several previous studies have looked into the fabrication of pectin-based films enriched with nano-compositions, such as sulfur nanoparticles, silver nanoparticles, and nano clays, for food and biomedical applications [28,29,30]. A distribution of nanomaterials within the film may enhance interface contact between film constituents and refine film performance [31]. However, there is limited research investigating pectin-based film incorporated with drug-loaded SLNs.

The main objective of the present study was to fabricate novel pectin mucoadhesive films incorporated with FZ-loaded SLNs (FZ-SLNs) for drug delivery through the buccal mucosa. High-DE pectin with a 70% DE and molecular weight of 200 kDa was employed as a mucoadhesive film-forming polymer. According to previous work conducted by our research group on the preparation of FZ-SLNs, Tween^®^ 80 and glyceryl monostearate (GMS) are potential ingredients that can form the FZ-loaded solid lipid particle at the nanoscale level [13]. Hence, Tween^®^ 80 and GMS were suitable for use as stabilizers and solid lipids, respectively. The dispersion of FZ-SLNs in pectin solutions was characterized. After the film preparation, the FZ-SLN-incorporated films were characterized in terms of thickness, mucoadhesion, mechanical characteristics, Fourier-transform infrared (FTIR) spectrum, drug release profile, and permeability study. In addition, the antifungal activity of the film samples against *Candida albicans* was also determined.

## 2. Results and Discussion

### 2.1. The Particle Characteristic of FZ-SLNs in Pectin Solutions

To assess the effect of the amount of film-forming polymer on the particle characteristics of FZ-SLNs in dispersions, the amounts of pectin were varied as described in formulations C1–C3 (Table 1), while the content of the stabilizer and solid lipid in the formulations was fixed.

**Figure 1:**
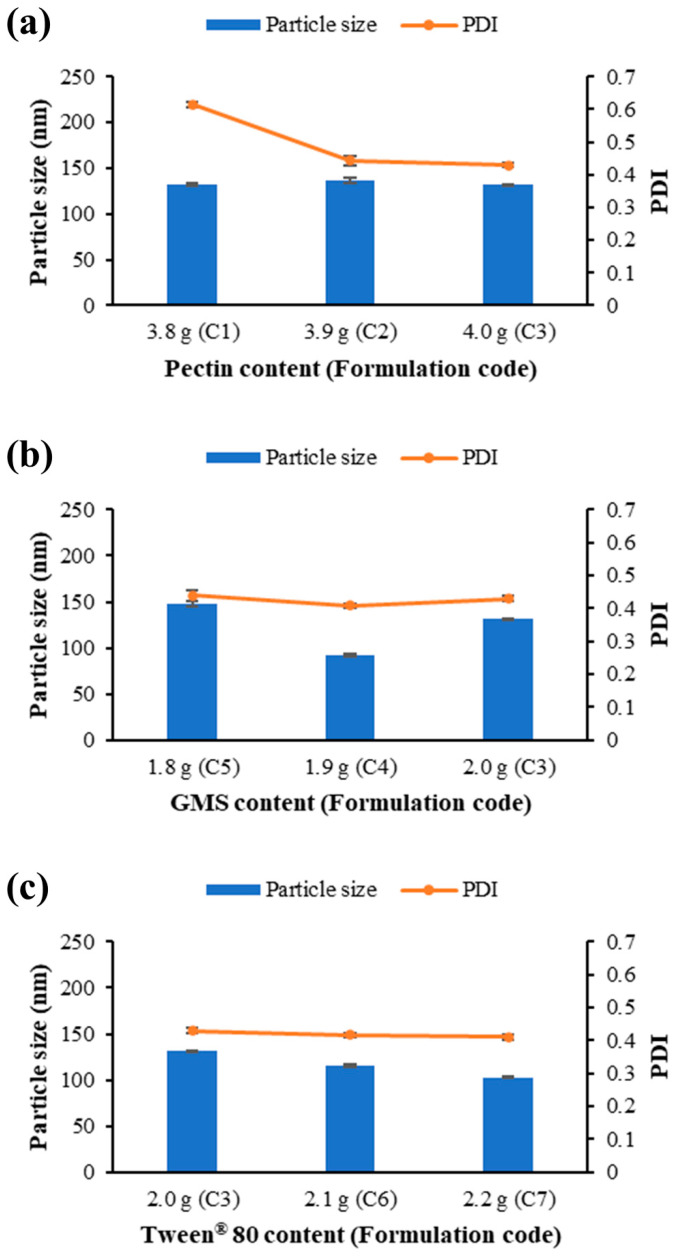
Particle size and polydispersity index (PDI) of FZ-SLNs in pectin solutions containing different content of (**a**) pectin, (**b**) GMS, and (**c**) Tween^®^ 80.

Figure 1a shows the particle size and polydispersity index (PDI) for FZ-SLNs in pectin solutions with different pectin content. As can be seen, the mean values of the particle size of FZ-SLNs were in the range between 131.7 and 136.6 nm, which were in the nanosize range. The small mean PDI values of FZ-SLNs in C2 and C3 were 0.444 and 0.430, respectively, while C1 represented a high mean PDI value of 0.615. The PDI values found in C2 and C3 were significantly lower than that of C1 (*p* < 0.05). The PDI values decline with increasing pectin content, indicating that the addition of pectin narrowed the particle size distribution. The small PDI value of 0.5 and below is acceptable and a representation of uniform SLN dispersion with a narrow particle size distribution [32,33]. The homogeneous size distribution leads to the desired effect on the physical stability of the nanoparticle dispersion and is a favorable particle characteristic [34]. Because the formulation C3 contained high mucoadhesive polymer content and possessed nanoparticles of a small size and a small PDI value, the pectin content as described in the formulation C3 was selected, and the content of solid lipid and stabilizer was varied further to investigate the effect of solid lipid and stabilizer contents.

As described in Table 1, the content of GMS was varied in formulations C3, C4, and C5. Figure 1b shows the particle size and PDI for FZ-SLNs in pectin solutions with different GMS content. The differences in the particle sizes among formulations C3, C4, and C5 were statistically significant. The formulation C4 contained the smallest particles compared to other formulations with distinct lipid contents (C3 and C5). This indicated that a decrease in the particle size of the SLNs was observed as the lipid content increased to a certain value, after which an increase in the particle size of the SLNs was observed. Although the increase in lipid concentration generally produces the size increment of SLNs, the reduction in particle size caused by an increase in lipid content has also been observed [35]. It was reported that a concentration-dependent increase in particle size began as the lipid content of the SLN dispersion exceeded a critical value because of the insufficient amount of the coating stabilizer layer for high lipid content above a certain level [35,36]. At lipid contents lower than the critical level, the increase in lipid concentration exerts a negligible influence on the size increment of particles [35].

As described in Table 1, the content of the stabilizer, Tween^®^ 80, was varied and increased in the formulations C3, C6, and C7. From Figure 1c, it can be seen that the mean values of the particle size decreased with increasing the content of Tween^®^ 80. The differences in the particle size values among these sample formulations were statistically significant (*p* < 0.05). The PDI values of the samples tended to decrease when the amount of stabilizer was increased. The tendency of the stabilizer to form a stabilizer layer at the interface and decrease the interfacial tension may prevent the agglomeration of lipid particles, leading to the formation of small particles with a uniform size distribution [32].

Formulations C3, C4, C6, and C7, which possessed an acceptable PDI and were the top four formulations with the smallest particle size, were selected for film preparation and subsequent film testing.

### 2.2. The Mucoadhesion of Pectin Films Containing FZ-SLNs

The films containing FZ-SLNs were successfully prepared using the film casting method for formulations C3, C4, C6, and C7. The FZ-SLN-incorporated film types obtained from formulations C3, C4, C6, and C7 were referred to as FC3, FC4, FC6, and FC7, respectively. The thickness values of the obtained films for FC3, FC4, FC6, and FC7 were 0.194 ± 0.012, 0.193 ± 0.013, 0.186 ± 0.005, and 0.183 ± 0.037 mm, respectively. There was no statistical significance in the thickness values among these obtained films.

**Figure 2:**
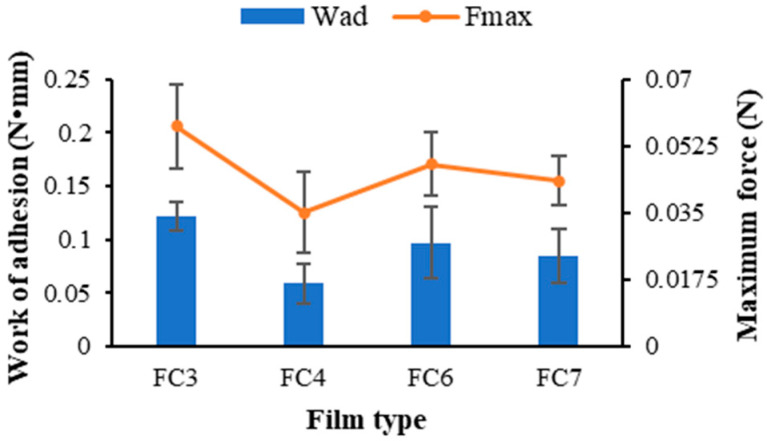
Mucoadhesive properties of prepared pectin films containing FZ-SLNs.

The mucoadhesion of FC3, FC4, FC6, and FC7 is shown in Figure 2. All films possessed the mucoadhesive ability. FC3 showed the greatest mean values for both work of adhesion (W_ad_) and maximum force (F_max_). However, there was no statistically significant difference in F_max_ values among FC3, FC4, FC6, and FC7. Also, the W_ad_ values of FC6 and FC7 were comparable. Although FC3, FC6, and FC7 contained distinct Tween^®^ 80 contents, there was no statistically significant difference in the W_ad_ values among these films. This may imply that the amount of Tween^®^ 80 had no discernible effect on the mucoadhesive property of the studied films. However, the W_ad_ values of the film formulations with high GMS content (FC3) were significantly greater than those of the film with low GMS content (FC4) (*p* < 0.05). The W_ad_ value reflects the adhesion energy between the mucoadhesive film and buccal mucosa. The structure of GMS contains hydroxyl groups, and pectin chains possess abundant carboxyl groups [15,23]. These functional groups play a crucial role in their interactions with mucin. Furthermore, the mucoadhesive characteristic of pectin may arise from their adsorption onto the mucin layer [37]. Therefore, using pectin as the film-forming polymer provided the FZ-SLN-incorporated film with the mucoadhesive ability. In addition, high GMS content in the FZ-SLN-incorporated film enriched the capacity for mucoadhesion compared with that of the film containing low GMS content.

### 2.3. The Mechanical Properties of Pectin Films Containing FZ-SLNs

**Figure 3:**
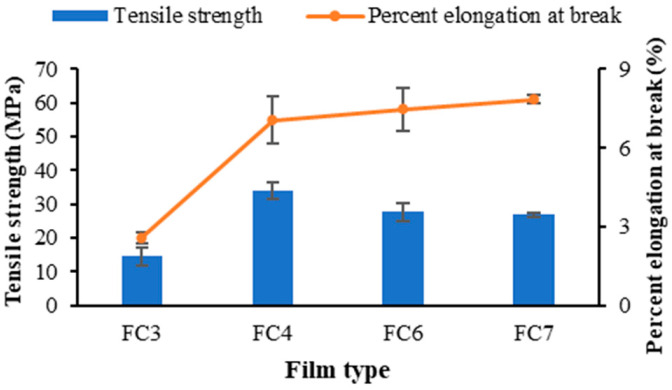
Mechanical properties of prepared pectin films containing FZ-SLNs.

Tensile strength is a quantitative mechanical parameter representing film integrity, and the percent elongation at break is a measurement of film flexibility [38,39]. Figure 3 represents the tensile strength and percent elongation at the break of FC3, FC4, FC6, and FC7. The tensile strength of FC4 was significantly greater than that of other films, while the values of tensile strength and percent elongation at the break of FC3 were significantly lower than those of others (*p* < 0.05). Based on the particle size of the FZ-SLNs dispersed in the studied film types, FC4 had the smallest nanoparticles, while FC3 contained the largest nanoparticles. The impact of the particle size variation on the mechanical characteristics of polysaccharide-based films containing nanoparticles has been reported by de Moura et al. [40]. The high surface area of small nanoparticles accelerated their interaction with the polysaccharide matrix during film formation, while the large nanoparticles caused the hindrance effect on film formation. Therefore, the presence of small nanoparticles caused higher film integrity and tenacity compared with film containing nanoparticles of a large size [40].

### 2.4. FTIR Spectra

**Figure 4:**
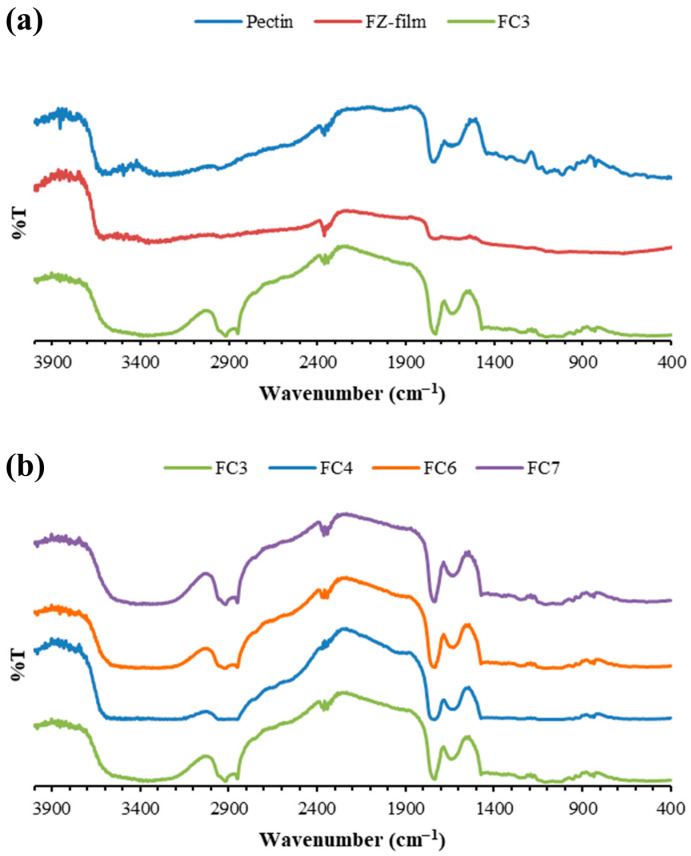
FTIR spectra of (**a**) pectin, FZ film, and FC3 and (**b**) distinct pectin films containing FZ-SLNs.

The FTIR spectra of pectin, FZ-loaded pectin film without SLNs (FZ film), and pectin films with FZ-SLNs are presented in Figure 4. As shown in the FTIR spectrum of pectin (Figure 4a), the two broad bands in the region between 3000 and 3700 cm^−1^ correspond to the stretching vibrations of hydroxyl groups, which are the abundant functional groups found in the chain structure of pectin [41,42]. In this OH stretching range, the band observed in the higher wavenumber region of 3450–3700 cm^−1^ reflects the presence of free OH groups, while the band appearing in the lower wavenumber region of 3000–3450 cm^−1^ corresponds to the existence of hydrogen-bonded OH groups [42]. The appearance of C=O peaks at 1629 cm^−1^ and 1739 cm^−1^ may be attributed to the presence of free and esterified carboxyl groups in the pectin chain structure [43]. The main characteristic peaks of pectin were also observed in FZ film (Figure 4a). According to the FTIR spectrum of FC3 (Figure 4a), two distinct peaks near 2848 cm^−1^ and 2914 cm^−1^, which can be assigned to CH stretching of GMS, revealed the presence of GMS in the film [15]. Although there was no noticeable change in the other main characteristic peaks of pectin in the spectrum of FC3, the band of the free OH groups seemed to have disappeared, and a band that broadened in the OH stretching range (3000–3700 cm^−1^) was observed in the FTIR pattern of FC3. The decrease in the sharpness and the broader band of OH stretching could relate to the formation of hydrogen bonds between the film matrix and nanocomponent [44]. Accordingly, the interaction between the pectin-based matrix and the composition of SLNs may cause a reduction in the number of free OH groups [44,45]. No apparent pattern alteration in the FTIR pattern of pectin films incorporating FZ-SLNs was observed when comparing the FTIR spectrum of FC3 with the spectra of various pectin films containing FZ-SLNs (Figure 4b).

### 2.5. Differential Scanning Calorimetry

**Figure 5:**
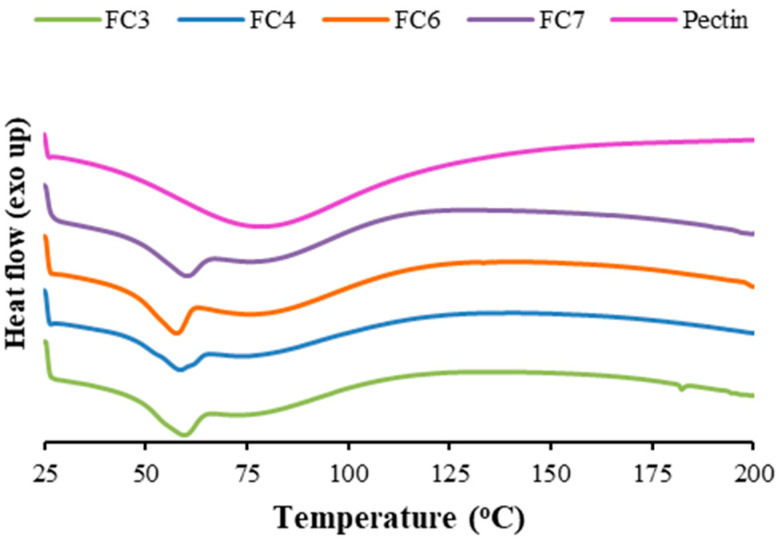
DSC thermograms of pectin and pectin films containing FZ-SLNs.

Figure 5 depicts the thermograms of pectin and pectin films with FZ-SLNs, which were characterized by differential scanning calorimetry (DSC). For pectin, the broad endothermic peak detected within the temperature range of 50 to 125 °C may reflect the release of water associated with the hydrophilic residues of the polymer. This thermal event has also been observed for pectin powder, as reported in the literature [46,47]. All pectin films with FZ-SLNs also exhibited this broad endothermic signal, but their thermograms displayed an additional endothermic peak at the low-temperature side in the range of 50 to 70 °C. The melting transition of GMS has been reported to be approximately 61.8 °C [13]. The presence of the endothermic signal in the range of 50 to 70 °C revealed the melting of GMS in the formulations upon heating. Although it has been reported that the plain FZ powder exhibited a sharp characteristic melting peak around 141 °C [13], the DSC thermograms of all pectin films with FZ-SLNs do not show the sharp melting peak of the drug. The integration of drug molecules into the carrier and the resulting conversion of the drug from a crystalline to an amorphous state may account for the lack of a drug melting peak in FC3, FC4, FC6, and FC7 [13]. All thermal events observed in pectin films with FZ-SLNs occurred at temperatures higher than 37 °C, suggesting that all formulations are thermally stable and maintain their solid state at body temperature.

### 2.6. In Vitro Release of FZ

**Figure 6:**
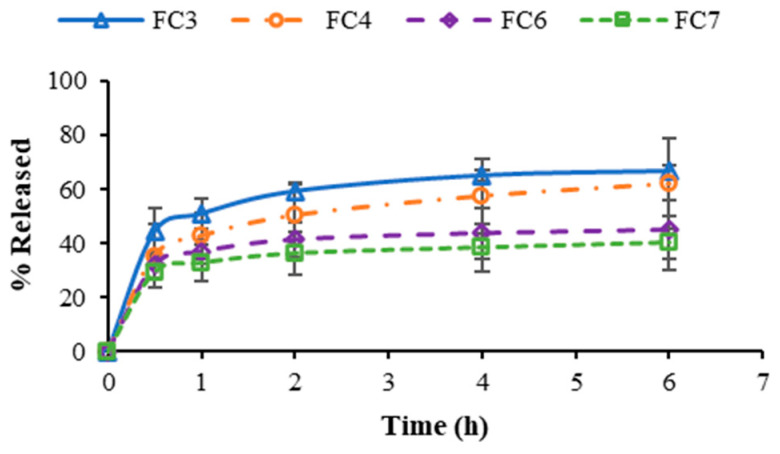
In vitro release of FZ from prepared pectin films containing FZ-SLNs.

The in vitro release profiles of FZ from pectin films containing FZ-SLNs are depicted in Figure 6. For all FZ-SLN-incorporated film types, the drug release patterns represented the burst release of FZ that occurred in the early stage and was after that followed by a sustained release over time. The lipophilicity of FZ is moderate, and FZ demonstrates viability in both aqueous and lipophilic environments [48,49]. Therefore, some FZ molecules may deposit at the surface of SLNs and the polymer matrix during preparation. The presence of free drug molecules in the outer layers of SLNs may account for the initial burst release of the drug from the SLN-loaded matrix, while the subsequent sustained release of the drug could be attributed to the liberation of the encapsulated drug molecules from the internal lipid phase [50,51]. The SLN structure as well as the polymeric matrix act as barriers to drug diffusion and transportation, resulting in sustained drug release [51]. The sustained drug release pattern with the burst in the initial stage could be beneficial in reducing the lag period of drug release [50,52]. The release patterns of FZ from FC6 and FC7 were comparable and showed a more sustained drug release than those from FC3 and FC4. Considering the presence of a surfactant in each FZ-SLN-loaded film type, the amount of Tween^®^ 80 in the films FC6 and FC7 was higher than in FC3 and FC4. It has been proposed that an inverse relationship between the Tween^®^ 80 content and the order of drug release occurs because a higher surfactant content stabilizes the SLNs and retards the release of the encapsulated drug from the lipid core to the outside of the SLNs [53].

### 2.7. Permeation Study

**Figure 7:**
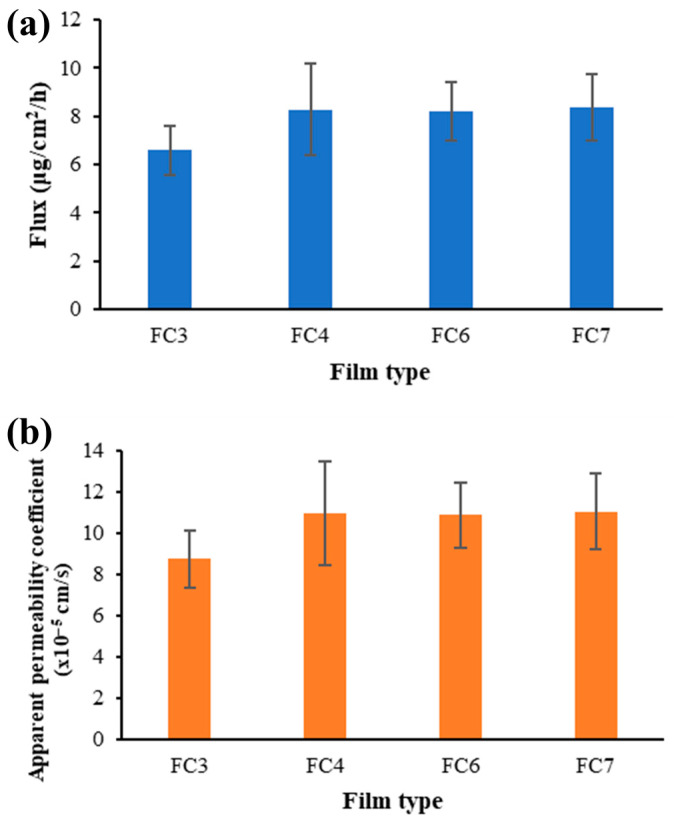
Permeability parameters, (**a**) flux and (**b**) apparent permeability coefficient, for prepared pectin films containing FZ-SLNs.

The permeability parameters, like the flux and apparent permeability coefficient for FC3, FC4, FC6, and FC7, are represented in Figure 7. Although there is no statistical difference among different film types for both parameters, the mean values of both parameters were found to be the lowest for FC3, which contained the largest size of FZ-SLNs. The mean values of the permeability parameters were found to slightly increase for FC4, FC6, and FC7, which possessed smaller FZ-SLNs. The variation in the permeability of various samples could be related to the size of the nanoparticles. The flux of drug-loaded SLNs across the mucosal membrane likely varies in relation to particle size, and drug carriers with smaller particle sizes provide good permeation ability [54].

### 2.8. In Vitro Antifungal Activity

**Figure 8:**
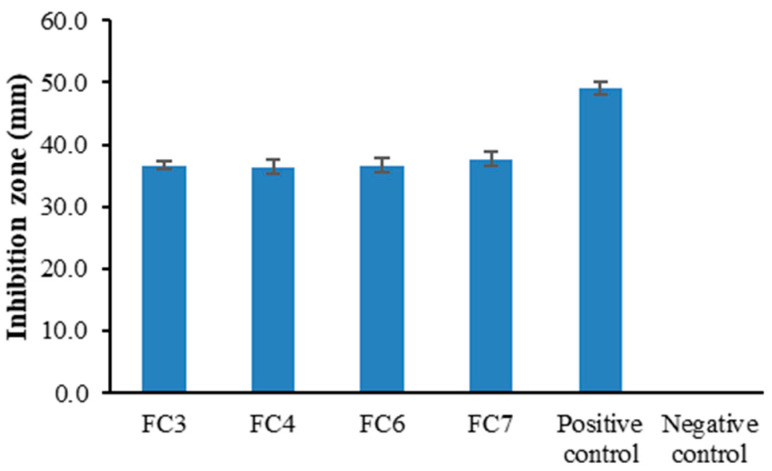
Diameters of the inhibition zone of prepared pectin films containing FZ-SLNs and controls.

The antifungal activity of the prepared FZ-SLN-incorporated films was investigated. Ethanol solution and the solution of FZ in ethanol were utilized as the negative control and positive control, respectively. The diameters of the inhibition zones are shown in Figure 8. The positive control showed antifungal activity, while no antifungal activity was observed for the negative control due to the lack of FZ in the negative control. All prepared FZ-SLN-incorporated films exhibited antifungal activity against *Candida albicans*. The diameter of the inhibition zone was virtually greater for the positive control compared to all prepared films. This seems to be attributed to the sustained release behavior of the prepared FZ-SLN-incorporated films, as described in the previous section, while the positive control contained FZ in the already-dissolved form in ethanol [15]. It has been reported that antifungal-loaded vehicles have similar but slightly lower in vitro antifungal activity when drugs are released from sustained drug delivery systems compared to the pure drug in media, and the antifungal activity could account for the release of drug molecules from carriers [15,55].

## 3. Materials and Methods

### 3.1. Materials

FZ was purchased from TCI (Tokyo, Japan). High-DE pectin with a 70% DE and molecular weight of 200 kDa was kindly provided by Herbstreith & Fox GmbH (Werder, Germany). GMS and Tween^®^ 80 (polysorbate) were purchased from PC Drug Co., Ltd. (Bangkok, Thailand). *Candida albicans* ATCC 10231 was purchased from the Department of Medical Sciences (Bangkok, Thailand). All additional chemicals employed in this investigation were of analytical grade and were utilized without any modifications.

### 3.2. Preparation of FZ-SLNs in Pectin Solutions and Pectin Films Containing FZ-SLNs

Table 1 shows the amounts of pectin, Tween^®^ 80, and GMS in each formulation of FZ-SLNs in pectin solution. The amounts of the drug (FZ) and glycerol used as a plasticizer were 0.075 g and 2 g, respectively, for all formulations. The remaining weight of the composition after subtracting the quantities of the ingredients mentioned above from the total formulation weight was the weight of the water used to prepare the formulation, with a total weight of 150 g.

The polymer solution was composed of pectin and glycerol. The required amount of pectin was dispersed in 3/4 of the water content of the formulation. After the homogenous pectin solution was obtained, 2 g of glycerol was dissolved in the pectin solution.

The FZ-SLNs were prepared using the hot homogenization method. To prepare the water phase, Tween^®^ 80 was dissolved in the remaining 1/4 of the water content, and the water phase was heated to 80 °C. The oil phase consisted of 0.075 g of FZ and GMS regarding the content described in Table 1 for each formulation. GMS and FZ were both melted and homogenized to prepare the oil phase at 80 °C. To prepare the primary emulsion, the oil phase was poured into the water phase, and both phases were mixed using a high-shear mixer at 7000 rpm for 5 min. The size of the internal phase was then reduced under ultrasonic amplitude at 100% by using an ultrasonic probe sonicator. During ultrasonication for 15 min, the sample was kept in an ice bath to acerate the formation of FZ-SLNs.

The FZ-SLNs in pectin solutions were fabricated by dispersing the prepared FZ-SLNs into the polymer solution with continuous mechanical stirring at 800 rpm for 30 min.

For preparing the pectin films containing FZ-SLNs, the FZ-SLNs in the pectin solution were transferred into the glass plate and dried according to the film casting method as previously described [15].

The FZ in the polymer solution for preparing FZ-loaded pectin film without SLNs (FZ film) was prepared by dissolving the same amount of FZ in 7.5 mL of ethanol and then adding the ethanol solution containing FZ to the polymer solution. FZ film was also obtained by using the film casting method.

### 3.3. The Particle Size and the Nanoparticle Size Distribution Characterization

The particle size and the nanoparticle size distribution reported as PDI were evaluated using dynamic light scattering. Each sample of FZ-SLNs in the pectin solution was diluted with distilled water before investigation using a Malvern Zetasizer Nano ZS (Malvern Instruments, Malvern, Worcestershire, UK) to prevent multi-scattering effects [15]. The experiments were conducted in triplicate.

### 3.4. Mechanical Characterization and Mucoadhesion Studies

A Mini Test 600 thickness gauge was employed to determine the thickness of the obtained films. Each prepared film was cut into a square shape with a size of 2.0 cm × 2.0 cm. The mechanical properties, including tensile strength and percent elongation at break, of the films were determined by using a TA-XT plus texture analyzer (Stable Micro Systems, Godalming, Surrey, UK). Porcine buccal mucosa was utilized as a biological membrane in the present work, owing to its resemblance to human buccal tissue [21]. The TA-XT plus texture analyzer fitted with a mucoadhesive holder was also used to measure the mucoadhesive characteristics according to the method of Kraisit et al. [21].

### 3.5. FTIR Spectroscopy

The FTIR spectra of plain pectin, FZ film, and pectin films containing FZ-SLNs were obtained using an FTIR spectrometer (IRTracer-100, Shimadzu, Kyoto, Japan). The samples were prepared using the KBr method. Each sample was ground, mixed with KBr powder, and compressed using a hydraulic press. The sample disc was placed in the sample holder and scanned from 4000 to 400 cm^−1^ at a resolution of 4 cm^−1^, with 20 scans.

### 3.6. DSC Measurements

DSC thermograms of plain pectin and pectin films containing FZ-SLNs were obtained in the temperature range of 25–200 °C with a heating rate of 10 °C/min, using a differential scanning calorimeter (DSC 3+, Mettler Toledo, Viroflay, France). The DSC scans were performed under a nitrogen atmosphere at a gas flow rate of 50 mL/min.

### 3.7. In Vitro Drug Release Study

The release patterns of FZ from the pectin films containing FZ-SLNs were determined in triplicate utilizing a modified Franz-type diffusion cell. The films with a circular shape and a diameter of 1.5 cm were placed on a fiber mesh before being applied to the receptor cell. A mixture of simulated salivary fluid (pH 6.8) and ethanol at a volume ratio of 1:1 was used as the release medium. The receptor chamber was filled with 15 mL of the release medium, and the temperature of the diffusion cell was controlled using a water jacket at 37 °C. At each time point, 0.5 mL of the release medium was taken and replaced with an equivalent volume of the new medium. The quantity of FZ in the collected release medium was analyzed using high-performance liquid chromatography (HPLC) coupled with a C18 column at 260 nm UV detection. The mobile phase used for HPLC separation was methanol/water (40/60, *v*/*v*), and the flow rate was 0.8 mL/min.

### 3.8. In Vitro Permeation Study

The in vitro permeation study was performed in triplicate using a modified Franz-type diffusion cell. The experimental setup closely resembled that outlined in the section on in vitro drug release study, with the exception that porcine buccal mucosa was utilized as the barrier between the donor compartment and the receptor chamber for the permeation study. The flux (μg/cm^2^/h), and the apparent permeability coefficient (×10^−5^ cm/s) were estimated from the permeation data.

### 3.9. In Vitro Antifungal Activity

The agar diffusion method was employed to assess the in vitro antifungal activity of the chosen films against *Candida albicans* ATCC 10231, as Phaechamud and Mahadlek described [56]. The fungi were cultivated and preserved at 37 °C for 48 h using Sabouraud dextrose broth (Difco^TM^, Franklin Lakes, NJ, USA). Subsequently, the broth was diluted to achieve a turbidity level of approximately 10^8^ colony-forming units (CFUs) per milliliter. The inoculated medium was swabbed and spread on a Sabouraud dextrose agar plate (Difco^TM^). Every film was precisely sliced into a circular shape with a diameter of approximately 1.0 cm and carefully positioned on the surface of the inoculated medium. In this experiment, sterilized cylinder cups were meticulously placed on the surface of the inoculated medium. Each cylinder cup was then filled with the positive control, which consisted of FZ dissolved in ethanol, and the negative control, which consisted of an ethanol solution. The films and controls were incubated at 37 °C for 48 h. The diameter of the inhibition zone for antifungal activity was measured in millimeters (mm).

### 3.10. Statistical Analysis

The means and standard deviations of the results from the experiments conducted in triplicate were computed and reported. The statistical comparison was performed using the analysis of variance (ANOVA). Statistical significance was accepted at the 0.05 level.

## 4. Conclusions

In this study, FZ-SLNs in pectin solutions were formulated, and their particle characteristics were investigated. The high amount of pectin resulted in a decrease in the size of the obtained FZ-SLNs. Similarly, increasing the amount of Tween^®^ 80 also reduced the size of the FZ-SLNs when pectin was present in high quantities. A decrease in the particle size of the FZ-SLNs as the GMS content increased was observed, followed by an increase in the particle size when the GMS content exceeded a certain value. Four formulations with small particle sizes and an acceptable PDI were chosen to fabricate novel pectin films with FZ-SLNs. Each of the selected formulations had a mean particle size not greater than 131.7 nm and a mean PDI value below 0.5. Despite variations in the excipient composition within the film, comparable film thicknesses were achieved. All prepared films showed mucoadhesive ability; the presence of high GMS content in FZ-SLN-incorporated films tended to enrich the mucoadhesiveness. The mechanical characteristic investigation revealed the impact of the particle size variation on film integrity. Based on the FTIR spectra, hydrogen bonds might form between the pectin-based matrix and the SLNs’ components. The DSC thermogram of each pectin film containing FZ-SLNs did not show any thermal events below 37 °C, indicating that the formulation stayed solid at body temperature. According to the drug release patterns, a sustained drug release pattern with a burst in the initial stage for all films could be beneficial in reducing the lag period of drug release. The film with a smaller particle size of FZ-SLNs showed a slight increase in permeability across the porcine mucosa. All film samples possessed antifungal activity. These results reveal the potential application of pectin films containing FZ-SLNs for buccal drug delivery.

## Figures and Tables

**Table 1:** Formulation composition of FZ-SLNs in pectin solutions.

Formulation Code	Formulation Composition (g) *		
	Pectin	GMS	Tween^®^ 80
C1	3.8	2.0	2.0
C2	3.9	2.0	2.0
C3	4.0	2.0	2.0
C4	4.0	1.9	2.0
C5	4.0	1.8	2.0
C6	4.0	2.0	2.1
C7	4.0	2.0	2.2

* The amounts of FZ and glycerol used for each formulation are 0.075 g and 2 g, respectively.

## Data Availability

Data will be made available on request.
